# Geroprotective applications of oleuropein and hydroxytyrosol through the hallmarks of ageing

**DOI:** 10.1007/s11357-025-01697-4

**Published:** 2025-05-27

**Authors:** Anna Calabrò, Anna Aiello, Paula Silva, Calogero Caruso, Giuseppina Candore, Giulia Accardi

**Affiliations:** 1 Laboratory of Immunopathology and Immunosenescence, Department of Biomedicine, Neurosciences and Advanced Diagnostics, University of Palermo, 90134 Palermo, Italy; 2 Laboratory of Histology and Embryology, Department of Microscopy, School of Medicine and Biomedical Sciences (ICBAS), University of Porto (U.Porto), Rua Jorge Viterbo Ferreira 228, 4050-313 Porto, Portugal; 3 iNOVA Media Lab, ICNOVA-NOVA Institute of Communication, NOVA School of Social Sciences and Humanities, Universidade NOVA de Lisboa, 1069-061 Lisbon, Portugal

**Keywords:** Ageing, Geroprotectors, Hydroxytyrosol, Inflammageing, Oleuropein

## Abstract

Geroprotectors are compounds that target the underlying mechanisms of ageing to delay the onset of age-related diseases and extend both lifespan and health span. As ageing is driven by the accumulation of cellular damage, DNA instability, epigenetic changes, mitochondrial dysfunction, and chronic inflammation, the concept of geroprotection focuses on compounds that can mitigate these processes. Oleuropein (OLE) and its derivative hydroxytyrosol (HT), both phenolic molecules derived from *Olea europaea* (olive tree), have gained significant attention as potential geroprotectors due to their potent antioxidant and anti-inflammatory properties. These phytochemicals, central to the Mediterranean diet, activate key molecular pathways such as nuclear factor erythroid 2-related factor 2, reducing oxidative stress and modulating inflammatory responses. Through these mechanisms, OLE and HT help counteract inflammageing, a critical factor in age-related dysfunction. This review highlights the role of OLE and HT as geroprotective agents, emphasising their ability to target the hallmarks of ageing and their potential to improve health span by slowing the progression of age-related conditions. With proven efficacy in various biological models, these compounds represent promising tools in the ongoing search for strategies to enhance the quality of life in ageing populations.

## Background

Ageing is an inevitable and intricate process, shaped by a cascade of molecular and cellular events that gradually undermine the organism’s ability to maintain homeostasis. As these disruptions take hold, the integrity of cells and tissues deteriorates, manifesting in the deregulation of critical molecular pathways called hallmarks of ageing [1]. Among the most prominent signs of ageing are DNA damage, epigenetic changes, and dysfunctions in essential organelles such as mitochondria and lysosomes. These disturbances contribute to a breakdown in proteostasis, impaired autophagy, and the accumulation of damaged proteins and senescent cells (SCs). Recent research has broadened this framework, introducing new hallmarks of ageing, including altered RNA processing, dysregulated autophagy, and disruptions in the microbiome [2]. These insights continue to refine our understanding of the complex and multifaceted nature of ageing.

The cumulative effects of these traits not only degrade cellular function but also compromise tissue and organ health, ultimately leading to physical decline and the onset of age-related diseases (ARDs). In this context, the search for interventions that target these hallmarks and can slow or reverse aspects of ageing has gained increasing interest.

The concept of “geroprotection”, first introduced by Ilya Mechnikov in “The Prolongation of Life: Optimistic Studies” [3], has emerged as a focal point in ageing research, referring to the potential of certain compounds to act on molecular, cellular, or physiological biomarkers of ageing, extending both lifespan and health span of studied organisms [4–7]. A compound to be considered a “geroprotector” should delay the onset of ARDs and exhibit efficacy across multiple biological models, making it broadly applicable, including in humans [3, 4].

Oleuropein (OLE) and its derivative hydroxytyrosol (HT), both derived from the fruits and leaves of *Olea europaea Linn* (olive tree), have attracted significant attention as geroprotectors due to their ability to modulate key processes related to ageing [4, 8]. These phytocompounds, key components of the Mediterranean diet (MedDiet), possess potent antioxidant and anti-inflammatory properties. By activating pathways like nuclear factor erythroid 2-related factor 2 (Nrf-2) and reducing oxidative stress, OLE and HT exert protective effects on cellular health, mitigating damage caused by reactive oxygen species (ROS) and inflammatory mediators [1]. Their ability to modulate these inflammatory pathways further highlights their potential in counteracting inflammageing, a key driver of age-related dysfunction [9].

**Fig. 1:**
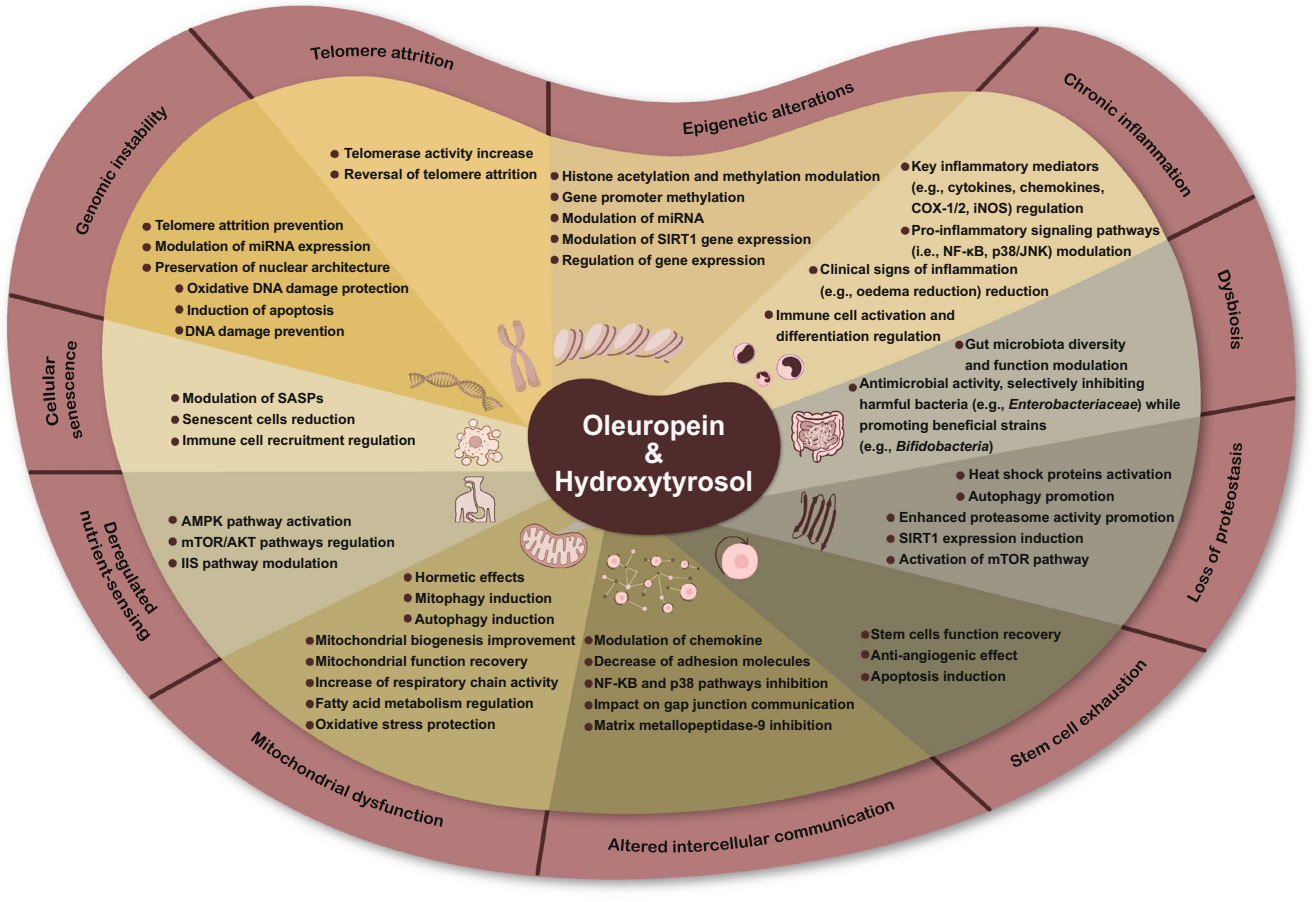
Mechanisms of action of oleuropein and hydroxytyrosol in hallmarks of ageing. This figure illustrates the various biological effects of OLE and HT in combating cellular ageing processes, targeting the hallmarks of ageing (i.e., genomic instability, telomere attrition, epigenetic alterations, loss of proteostasis, deregulated nutrient sensing, mitochondrial dysfunction, altered intercellular communication, stem cell exhaustion, cellular senescence, dysbiosis, and chronic inflammation)

This paper aims to analyse the role of OLE and HT in modulating the hallmarks of ageing described by Lopez-Otin et al. [1], exploring their mechanisms of action and their potential to enhance health span and positioning them as valuable tools in the ongoing effort to combat the detrimental effects of ageing. In this context, Fig. 1 summarises the contribution of OLE and HT on the hallmarks of ageing, based on the literature reviewed in this study; Tables 1, 2, and 3 summarise key in vitro and in vivo studies, including human trials, that highlight the role of OLE, HT and olive extracts in modulating the hallmarks of ageing.

**Table 1:** In vitro studies exploring the role of OLE, HT, and extracts from EVOO, OO, and olive leaf in modulating the hallmarks of ageing. References: [10–38]

Compound	Dose	Model	Time of treatment	Outcome	References
Genomic instability					
OLE	40–640 μM	Human leukaemia cell line (HL60)	72 h	Reduction of cancer cell viability	[10]
OLE	100 µM or 200 µM	Human breast cancer cell line (MCF-7)	72 h	Upregulation of pro-apoptotic and cell cycle checkpoint proteins; downregulation of anti-apoptotic proteins	[11]
OLE	100–1400 μg/ml	Human breast cancer cell line (MCF-7 and MDA-MB-231)	24 h; 48 h	Increased expression of pro-apoptotic genes; decreased expression of anti-apoptotic genes	[12]
OLE	0–800 µM; 0–400 µM	Human breast cancer cell line (MDA-MB-231 and MDA-MB-468)	48 h	Increased expression of pro-apoptotic genes; decreased expression of anti-apoptotic genes	[13]
OLE	100 µM	Bovine vascular smooth muscle cells	24 h	Induction of cell cycle block between the G1 and the S phases	[14]
OLE	20, 50, and 100 μM	Immortalised human proximal tubular epithelial cell line (HK2)	60 h	Induction of the demethylation of Nrf-2 and KLOTHO	[15]
Epigenetic alterations					
HT (3,4-dihydroxyphenylethanol, DOPET)	100 µM	Organotypic hippocampal slice cultures from mice	30 min	Decreased expression of HDAC6	[16]
OLE	600 μg/mL	Human breast cancer cell line (MCF-7)	24, 48, and 72 h	Reduced expression of HDAC2 and HDAC3	[17]
Phenolic extracts from EVOO	0.0–0.1% dissolved in ethanol	Human HER2-positive (HER2⁺) breast cancer cell line (JIMT-1)	5 days	Hyperacetylation of histone H3	[18]
HT	100 µM	Human primary and immortalised cell line of chondrocytes (C-28/I2)	4–24 h	Increased expression of miR-9	[19, 20]
HT	10 μM	Human epithelial colorectal adenocarcinoma cell line (Caco-2); intestinal epithelial cells	24 h	Modulation of miRNA related to apoptosis, proliferation, and lipid metabolism pathways	[21]
Cellular senescence					
OLEa or HT	OLEa 5 µM; HT 1 µM	Neonatal human dermal fibroblasts	2 weeks	Reduction of SA-β-Gal-positive cells; protection from DNA damage induced by senescence; maintenance of the integrity of the nuclear membrane; reduction of γH2 AX expression and cGAS/STING pathway activation	[22]
Telomere attrition					
OLE; oleacein	1, 2, 5, and 10 μM	Endothelial progenitor cells	3 h	Increased telomerase activity	[23]
Mytochondrial dysfunction					
Olive leaf extracts with 87% of OLE	200 μg/mL	Human breast cancer cell line (MDA-MB-231); human ovarian adenocarcinoma cell line (OVCAR-3)	24 h	Increased mitochondrial dysfunction, coupled with a decrease in functionality; increased ROS production; anti-tumour activity	[24]
HT	0.1–10 µmol/L	Mouse embryonic fibroblast cell line (3 T3-L1) to obtain adipocytes	48 h	Regulation of mitogenesis, β-oxidation factors, and fatty acids transport	[25]
OLE	1–50 μM	Pheochromocytoma of the rat adrenal medulla cell line (PC12) as model of Parkinson’s disease	24 h	Regulation of mitochondrial activity and biosynthesis; induction of apoptosis; regulation of membrane potential and ER post-translational activity (i.e., elimination of unfolded proteins)	[26]
Loss of proteostasis					
OLEa	50 μM	Human neuroblastoma cells (SH-SY5Y) and rat insulinoma cells (RIN-5 F)	4–24 h	Inhibition of mTOR/PKB-AKT pathway	[27]
OLE	100 μM	Human adipose-derived stem cells	24 h	Decreased LC3 levels; induction of mTOR, Beclin, and phosphorylation of AMPK	[28]
Stem cell exhaustion					
OLE	1, 2, 5, and 10 μM	Endothelial progenitor cells	3 h	Restoration of the capacity of migration and adhesion of endothelial progenitor cells in a concentration-dependent manner after treatment with AngII, which induces a senescent phenotype	[23]
OLE	10⁻⁶ to 10⁻^4^ M	Human bone marrow mesenchymal cells	21 days	Induction of osteoblastogenesis; protective effects against bone loss during ageing, including the inhibition of osteoporosis	[29]
HT	1/100 μmol	Human bone marrow mesenchymal cells	7–14 days	Increased adipogenic differentiation; inhibitory effects on osteogenic differentiation	[30]
Altered intercellular communication					
EVOO extracts	12.5 µg/mL	Murine dendritic cells	24 h	Reduction of TNF-α and IL-6 secretion, of CD86 expression, along with a down-modulation of IL-1β and iNOS expression	[31]
Altered cell motility					
OLE	0.01%	Fibroblasts and adherent tumour cell lines (LN-18, RPMI-7951, 786-O, and T-47D)	From 2 h to 10 days	Modification of the cytoskeleton and cellular motility capacity	[32]
Chronic inflammation					
HT	25, 50, and 100 μM	Human monocytic leukaemia cell line (THP-1)	3 h	Reduction of TNF-α, iNOS, and COX-2 protein and gene expression levels	[33]
HT	200 μM	Murine monocyte/macrophage cell line (J774)	24 h	Downregulation of iNOS and COX-2 gene expression through the inhibition of NF-κB, STAT-1α, and IRF-1	[34]
HT	100 μM	Freshly isolated human monocytes from healthy donors	24 h	Reduction of COX-2 and prostaglandin E2 expression; increase in TNF-α production	[35]
HT	0.25, 0.5, 1 μM	Peripheral blood mononuclear cells (PBMCs)	30 min–24 h	Reduction of cytokine secretion induced by a proinflammatory stimulus; inhibition of ROS production; inhibition of p38 phosphorylation controlling chronic immune and/or inflammatory processes	[36]
OLEa	0–50 μg/mL	Human THP-1 monocytic leukaemia cells (ATCC)	6 h	Decreased expression of MMP-9, TNF-α, and adhesion molecules (i.e., E-selectin, ICAM-1 and VCAM-1) via NF-κB modulation	[37]
OO extracts	6.25 μg/mL	PBMCs from obese children	6–24 h	Downregulation of CD16 expression; genes involved in the inflammatory response (e.g., PPARγ, CCL2, CCL4)	[38]

*OLE* Oleuropein, *Nrf-2* nuclear factor erythroid 2–related factor 2, *HT* hydroxytyrosol, *DOPET*: 3,4-diidrossifeniletanolo, *HDAC* histone deacetylase, *EVOO* extra virgin olive oil, *miR* microRNA, *miRNA* microRNA, *OLEa* oleuropein aglycone, *SA-β-Gal* senescence-associated β galactosidase, *ROS* reactive oxygen species, *ER* endoplasmic reticulum, *mTOR* mammalian target of rapamycin, *PKB* protein kinase B, *AMPK* 5'AMP-activated protein kinase, *AngII* angiotensin II, *TNF-α* tumour necrosis factor α, *IL* interleukin, *iNOS* inducible nitric oxide synthase, *COX-2* cycloxygenase 2, *NF-κB* nuclear factor kappa-light-chain-enhancer of activated B cells, *STAT-1* signal transducer and activator of transcription 1, *IRF-1* interferon regulatory factor 1, *PBMC* peripheral blood mononuclear cells, *MMP-9* matrix metalloproteinases, *ICAM-1* intercellular adhesion molecule-1, *VCAM-1* vascular cell adhesion molecule-1, *OO* olive oil, *PPAR* peroxisome proliferator–activated receptors

**Table 2:** In vivo studies on animal models exploring the role of OLE, HT, and extracts from EVOO, OO and olive leaf in modulating the hallmarks of ageing. References: [21, 39–49]

Compound	Dose	Model	Time of treatment	Outcome	Reference
Epigenetic alterations					
HT	1.5 mg/kg of feed per day	Sows as a model of pregnancy and to study the effects of diet supplementation on the foetus	65 days	Reduced impact of oxidative stress on DNA hypomethylation; increased methylation of whole DNA	[39]
OLE	50 mg/kg of diet	Transgenic hemizygous CRND8 male and female mice, carrying a double-mutant form of the human APP695 gene as a model of Alzheimer’s disease (ADs)	8 weeks	Enhancement of histone acetylation at H3 K9 and H4 K25, resulting in protection against ADs; reduction of HDAC2	[40]
HT	0.03 g% HT	C57BL/6 mice as a model of dietary intervention	8 weeks	Modulation of miRNA related to apoptosis, proliferation and lipid metabolism pathways	[21]
Deregulated nutrient-sensing pathways					
OLE	20 mg/kg	Diabetic rabbits	16 weeks	Reduced glucose levels; decreased oxidative stress; restoration of anti-oxidative systems; improved insulin sensitivity	[41]
OLE and HT	8–16 mg/kg of body weight	Diabetic rats	4 weeks	Reduced blood glucose levels; increased glycogen levels; reduction of triglyceride concentration; increased anti-oxidative enzyme activity; increased scavenger activity	[42]
Loss of proteostasis					
OLE	50 mg/kg of diet	Transgenic hemizygous CRND8 male and female mice, carrying a double-mutant form of the human APP695 gene as a model of ADs	8 weeks	Reduction of ß-amyloid deposition; favour preformed plaque disassembly; triggering autophagic machinery leading to the delivery of autophagosome substrates to lysosomes for degradation	[43]
Olive leaf extracts containing OLE	695 μg/kg body weight/day, containing 375 μg/kg body weight/day of OLE	Mouse model of ADs	3 months	Reduction of β-amyloid deposition; favour preformed plaque disassembly; activation of Nrf-2; reduction of neuroinflammation via inhibition of NLRP3 inflammasome and NF-κB activation; increased expression of adhesion molecules (e.g., VE-cadherin)	[44]
OLE	3% of OLE dissolved in drinking water	C57BL/6 J mice as a model of non-alcoholic fatty liver disease	8 weeks	Counteraction of unhealthy diet-related liver steatosis by inducing autophagy and mTOR activation	[45]
HT	100, 250, and 500 mg/kg	Rats as a model of acute inflammation	4.5 h	Inhibition of acute inflammation; decrement of IL-1β and TNF-α; lack of increase in IL-10 expression	[46]
HT	0.5 and 5 mg/kg	Rodent model of rheumatoid arthritis	12 days	Decreased oedema formation; decreased COX-2 and iNOS expression; reduced bone resorption, soft tissue swelling, and osteophyte formation, improving articular function	[47]
OLE	200 mg/kg	Diabetic mice	15 weeks	Reduction of blood glucose levels; improved glucose tolerance; reduction of the HOMA-IR index; promotion of hepatic PKB activation	[48]
Stem cell exhaustion					
HT	100 mg/Kg per day	Btg1 knockout and wild-type mice (C57BL/6 background) as a model of ageing neurodegeneration	30 days	Stimulation of the proliferation of stem and progenitor cells; inhibition of neurogenesis decline with ageing	[49]

*HT* Hydroxytyrosol, *OLE* oleuropein, *ADs* Alzheimer’s disease, *HDAC* histone deacetylase, *miRNA* microRNA, *Nrf-2* nuclear factor erythroid 2–related factor 2, *NF-κB* nuclear factor kappa-light-chain-enhancer of activated B cells, *mTOR* mammalian target of rapamycin, *IL* interleukin, *TNF* tumour necrosis factor, *COX-2* cycloxigenase 2, *iNOS* inducible nitric oxide synthase, *HOMA-IR* homeostatic model assessment for insulin resistance, *PKB* protein kinase B

**Table 3:** Human studies exploring the role of OLE, HT, and extracts from EVOO, OO and olive leaf in modulating the hallmarks of ageing. References: [21, 50]

Compound	Dose	Time of treatment	Clinical trial	Sample size	Age in years (age ± SD)/age range in years	Sex	Outcome	Reference
Deregulated nutrient-sensing pathways								
Olive leaf extract	4 capsules/day with OLE 51.1 mg and HT 9.7 mg or placebo	12 weeks (6 weeks of treatment followed by washout, after which participants crossed to the opposite intervention)	Randomised, double-blinded, placebo-controlled, crossover trial	46	46.4 ± 5.5	Men	Improvement of insulin sensitivity in the treated group; improvement in pancreatic β cell responsiveness; increased fasting IL-6, IGFBP1/2 concentrations	[50]
Epigenetic alterations								
HT	1 capsule with 25 mg/day of HT or placebo	1 week with placebo followed by 1 week with treatment	Randomised, double-blind and placebo-controlled trial	21	20–40	Men	Modulation of miRNA related to apoptosis, proliferation and lipid metabolism pathways	[21]

*SD* standard deviation, *OLE* oleuropein, *HT* hydroxytyrosol; *IL* interleukin, *IGFBP* insulin-like growth factor binding protein, *miRNA* microRNA

This review naturally continues the research conducted under the European Horizon 2020 ISOLDA project (Improved Vaccination Strategies for Older Adults, grant agreement No. 848166), which aims to develop enhanced vaccine formulations enriched with natural compounds like OLE [51]. These formulations are designed to address the challenges of efficacy and side effects arising from the molecular and cellular alterations associated with ageing, specifically by reducing baseline chronic inflammation and oxidative stress, key factors contributing to the reduced effectiveness of vaccines in the majority of the older population.

## Effects of OLE and HT on genomic instability

Genomic instability is the accumulation of genetic damage throughout life. Usually, the cell responds to damage by arresting the cell cycle and, consequently, repairing the damage, inducing programmed cell death (apoptosis), or entering senescence.

Several studies have highlighted the role of polyphenols in preserving genomic instability. The mechanisms through which they appear to act involve protecting against potential DNA damage, such as mutations affecting genome-protecting genes (e.g., p53), reducing DNA double-strand breaks, and regulating the expression of genes involved in apoptosis or DNA architecture (e.g., histones and lamins) [52, 53].

Polyphenols influence key DNA repair pathways, including base excision repair, nucleotide excision repair, and double-strand break repair. For example, epigallocatechin gallate from green tea and resveratrol from grapes have been shown to enhance the expression of DNA repair proteins, including ataxia-telangiectasia mutated (ATM), BRCA1, and RAD51, which facilitate the efficient repair of DNA lesions and maintain chromosomal stability [52, 53]. There is a lack of direct evidence for OLE and HT in modulating genes involved in DNA damage repair mechanisms. They effectively neutralise ROS, thereby reducing oxidative DNA damage [54, 55]. Indeed, a complex mixture of phenols extracted from virgin olive oil and olive mill wastewater, along with HT and OLE derivatives, effectively protects DNA from hydrogen peroxide–induced damage, demonstrating potent scavenging activity [56, 57]. Additionally, OLE and HT safeguard mitochondrial DNA (mtDNA) from oxidative stress–induced damage and modulate ROS production associated with mitochondrial dysfunctions in several studies about in vitro cancer models and ARDs’ models [24, 58]. Damage to mtDNA affects its function, leading to reduced energy availability for cells and increased ROS production [59]. The primary threat to mtDNA is ROS accumulation, which results from excessive production or insufficient clearance, the absence of histones for protection, and an inefficient proofreading mechanism of γ-polymerase, the enzyme responsible for mtDNA replication [60]. The accumulation of defective mitochondria is consequently associated with other hallmarks, such as altered autophagy and loss of proteostasis [1]. The accumulation of damaged organelles leads the cell towards senescence and ARDs [60].

Along with the protection from DNA damage induced by oxidative stress, OLE, and HT induce apoptosis, demonstrated in in vitro models of cancer. The treatment of promyelocytic cancer cell lines (i.e., HL60, MCF-7, MDA-MB-468 cells) with OLE showed a dose-dependent reduction of cancer cell viability, primarily through the induction of apoptosis. This effect was associated with an upregulation of pro-apoptotic (e.g., Bax, caspase) and cell cycle checkpoint proteins (e.g., p53), alongside with a downregulation of anti-apoptotic ones (e.g., Bcl-2) [10–13]. In other cancer or vascular smooth muscle cell models, OLE specifically induces the expression of p53, acting via the extracellular signal-regulated kinase 1/2 pathway. This resulted in the arrest of the cell cycle in the G1-S and G2 phases [14, 61].

OLE and HT preserve DNA from damage by maintaining lamin B1 expression and reducing senescence associated secretory phenotype (SASP) production, mediated by nuclear factor-κB (NF-κB). This was demonstrated in a model of senescence induced by irradiating neonatal human dermal fibroblast. The downregulation of lamin B1 is indeed considered a marker of senescence [22]. The disruption of nuclear architecture is, indeed, one of the principal traits of genomic instability and the cause of progeroid syndromes (rare diseases with premature ageing manifestation), characterised by modification in the structure, production, and function of nuclear laminins, in particular of prolamin A, also called progerin. The dysfunction of the laminins also has effects on telomere attrition because of the role of the nuclear cytoskeleton to tether chromatin and the protein complexes associated with it [1].

## Effects of OLE and HT on epigenetic alterations

Epigenetic alterations are reversible heritable modifications, regulating the gene expression that occur without alteration of DNA sequence. The fine regulation of the epigenome determines whether, when, and where a gene is either silenced or expressed. These variations impact ageing at various levels. Recently, epigenome-wide association studies identified a lot of CpG markers whose methylation levels are strongly correlated with age, e.g., the promoter of the gene ELOVL2, KLF14, TRIM59, and FHL2, all included in the “Horvath epigenetic clock” [62, 63]. Generally, with age, there is a massive loss of constitutive heterochromatin, which influences the chromosome structures and, therefore, can be the cause of chromosomal changes. At the same time, a diffused hypomethylation of some gene loci was observed along with a decrease in trimethylation of histones, like H3 K9 and H4 K20, which were involved in the structure of chromosome extremity. This led to a consequent attrition of telomeres ending [64, 65]. Conversely, an increase in the hypermethylation of senescence-associated heterochromatin foci, which regulates the expression of genes involved in cell cycle checkpoints, was observed. This modification leads to the permanent arrest of SC proliferation. Notably, this phenomenon is closely associated with the silencing of cyclin A and is linked to the role of senescence in suppressing tumour cell proliferation. Specifically, it is a key feature of oncogene-induced senescence, a crucial mechanism in tumour suppression [66]. These changes result in a heterogeneity of the epigenetic pattern, creating a kind of epigenetic mosaic. Several authors proposed to combine these markers to formulate prediction models able to estimate the age of a subject [67–71]. Currently, the most robust methylation-based age prediction method is represented by the so-called “epigenetic clock”, formulated by Horvath [68]. Horvath’s epigenetic clock is a highly accurate tool for estimating both chronological age (the actual number of years a person has lived) and biological age (a measure of physiological ageing based on molecular markers, including epigenetic signature). It can be applied to almost any tissue and provides valuable insights into an individual’s overall health, including physical and cognitive fitness. Because biological age can either be lower or higher than chronological age, the clock can indicate whether a person is ageing more slowly or more rapidly than expected, offering a useful measure of their ageing process and health status [72, 73]. Indeed, by regressing DNA methylation age on chronological age, the epigenetic clock can determine whether biological age acceleration occurs in several diseases. Similar to the effect of ageing, several environmental stimuli, such as diet, education, and other social factors, correlate with the rate of epigenetic ageing. Moreover, interventions and manipulations, e.g., calorie restriction or treatment with specific molecules, increase lifespan and slow epigenetic ageing as well [74, 75].

The epigenetic modification occurring with ageing could be given also by alteration in enzymes involved in histone and chromatin modifications, like histone deacetylases (HDACs), methylase, or acetylases. Belonging to the class of deacetylases are the sirtuins, enzymes NAD-dependent, with a pleiotropic role in the ageing process. Sirtuin 1 (SIRT1), 3, and 6 are considered ageing markers, and their modulation was demonstrated to influence the prolongation of lifespan in animal models [76].

It has been demonstrated that OLE, or secoiridoids of extra virgin olive oil (EVOO) in general, can affect epigenetic modifications inducing histone modification through inhibition of HDAC2 and HDAC3 [77], and favouring hyperacetylation of H3 in cell cultures [18, 78]. In an Alzheimer’s model, OLE administration modified the acetylation of histones H3 K9 and H4 K25 and reduced the expression of HDAC2 [40]. The effects of OLE and HT are also evident in the modulation of genes involved in the metabolism of lipids and glucose or the inflammageing, determining the methylation or the acetylation of promoters. For example, in human placenta, the anti-inflammatory effects of epigenetic modification induced by OLE are manifested by increased H3 acetylation at forkhead box (FOX) P3, interleukin (IL)−10RA and IL-7R promoters and decreasing H3 K9 trimethylation, which consists in one of the major epigenetic modifications that occurs with ageing. In a study on MedDiet application, the modulation of expression of some enzymes, like stearoyl-CoA desaturase, was regulated by the methylation of the enzyme gene promoter, and this had effects on obesity, diabetes, and insulin resistance condition, impacting also on membrane composition in phospholipids of a HeLa cells [18, 48]. The treatment of sows with HT for 65 days showed a reduced impact of oxidative stress on DNA hypomethylation and so increased methylation of whole DNA [39]. HT had an impact on HDAC6, which acts on α-tubulin in an in vitro model of diabetes while, in an in vivo model, it operates oppositely [16]. Epigenetic modification induced by OLE and HT also influences the anti-tumour activity. Treatment of the MCF7 cell line with OLE glucoside led to a significant reduction in the expression of the HDAC2 and HDAC3 [17, 78]. In kidney cells treated with d-galactose to induce ageing, hypermethylation, which is driven by the upregulation of DNA methyltransferases 1, 3a, and 3b, lets to a significant suppression of Nrf-2 and KLOTHO, a key anti-ageing membrane protein predominantly expressed in the kidney [15]. OLE treatment of cultured cells effectively inhibited the epigenetic suppression of Nrf-2 and KLOTHO in a DNA methyltransferase-sensitive manner, demonstrating OLE’s strong DNA demethylation potential [15]. Furthermore, secoiridoids promote histone H3 acetylation through HDAC inhibitory activity, involving the p38 mitogen–activated protein kinase (MAPK) pathway [14].

Several studies conducted with EVOO extracts, comprising OLE and HT, resulted in a modulation of microRNA (miRNA) expression that influences the anti-inflammatory, anti-oxidant, and anti-tumoral effects of these molecules [78]. With ageing, the expression of some miRNA is dysregulated, and this impacts various aspects involved in the senescence process. Some miRNAs are considered ageing markers and may be considered one of the causes of genome instability, although they are considered part of the epigenome field [79].

In a model of oxidative stress in chondrocytes, HT was shown to determine the increased expression of miR-9, through the methylation of the promoter. This, in turn, influenced SIRT1 expression levels, which positively affects genome instability, metabolic efficiency, and mitochondrial formation and, instead, downregulates inflammatory mediators [1, 19, 20]. The effect of HT treatment on miRNA expression has been demonstrated both in vivo and in vitro models. In mouse models, HT administration significantly influenced the expression of key miRNAs involved in the regulation of lipid metabolism across multiple organs, including the liver, heart, adipose tissues, spleen, and brain [21]. Among these miRNAs, several were also found to be modulated in human cell cultures, reinforcing their relevance across species [21]. Furthermore, a 1-week HT supplementation (25 mg/day) in a randomised, placebo-controlled human trial led to a significant increase in miR-193a-5p expression in peripheral blood mononuclear cells, suggesting that HT can modulate miRNA expression both in animal models and in humans. miR-193a-5p, in particular, is involved in several biological processes, including lipid metabolism, cancer development, inflammation, and immune response [80].

Epigenetic mechanisms, including DNA methylation and miRNA modulation, play a central role in the pathophysiology of cardiovascular diseases (CVDs), and accumulating evidence supports the influence of EVOO and its polyphenols in this regulatory network. Dietary interventions using different formulations of EVOO, as well as adherence to a MedDiet or a low-fat diet, have been shown to modulate the expression of miRNAs involved in endothelial function and the inflammatory response, i.e., two critical processes in CVDs development. Notably, changes in DNA methylation patterns have also been observed, such as in the EEF2 gene, where increased methylation levels were positively correlated with pro-inflammatory biomarkers tumour necrosis factor (TNF)-α and C-reactive protein, suggesting a diet-driven epigenetic link to inflammation [81–83]. Let-7, a member of a conserved family of miRNAs with anti-inflammatory and anti-fibrotic properties, is one of the most studied miRNAs. Phenol-enriched EVOO has been shown to influence postprandial levels of circulating miRNAs, including let-7 miRNA, which are associated with cardiovascular protection [82]. Moreover, hydroxytyrosol-3-O-sulfate, a major metabolite of EVOO polyphenols, has been reported to prevent pathological endothelial-to-mesenchymal transition by preserving let-7 miRNA expression and suppressing transforming growth factor-β signalling activation, a key pathway in endothelial dysfunction [84]. Further, supporting the epigenetic impact of EVOO-rich diets, the PREvención con DIeta MEDiterránea (PREDIMED) study demonstrated that long-term adherence to a MedDiet modulates the expression of specific miRNAs, such as miR-410, which regulates the lipoprotein lipase gene variant rs13702. This modulation was associated with a reduced incidence of stroke over a median follow-up of 4.8 years, providing clinical relevance to diet-induced epigenetic changes in cardiovascular outcomes [85].

## Effects of OLE and HT on telomere attrition

The telomeres shortening is strictly associated with ageing and occurs principally in somatic cells. The alteration of telomerase activity or the modified expression of shelterin complex (i.e., specialised proteins that protect the ends of chromosomes) can cause telomere attrition that is linked to genomic instability. It also depends on oxidative stress and alteration in epigenetic modifications. Otherwise, it is linked to structural alterations of the nuclear lamina, with increased expression of progerin [1], representing a key factor in cellular ageing and senescence.

The role of OLE and HT in the contrast of telomere shortening is not yet discussed in the literature, but there are notions about the effect of MedDiet administration on telomere length [86]. The MedDiet is characterised by the assumption of EVOO as a dressing and a main source of fat [87]. Although OLE and HT are minor polar compounds in olives and much more represented in the leaves, their contribution to the beneficial properties of olive oil (OO) assumption is widely confirmed [88]. A 5-year treatment with MedDiet in the PREDIMED-NAVARRA study showed an increase in the telomere length in association with a decrease in anthropometric parameters associated with obesity [89]. Furthermore, the association between leukocyte telomere length and adherence to MedDiet showed a variable dependence related to the population taken into consideration, previously considering the leukocyte telomere length as a marker of ageing [90, 91]. These data about the positive role of MedDiet adherence, comprising OO assumption, were partially demonstrated by experiments on endothelial progenitor cells treated with OLE for 3 h, resulting in an increase in telomerase activity with decreased SCs and ROS formation [23].

## Effects of OLE and HT on mitochondrial dysfunction

Mitochondria are involved in several cellular functions related to ageing. They participate in the regulation of cellular survival through apoptosis induction, energy maintenance, and metabolic activity. As mentioned earlier, mtDNA may be interested in mutations determined by lack of γ-polymerase proof-reading activity, lack of DNA-associated proteins, and DNA damage induced by oxidative stress [1]. This impacts the expression of mitochondrial proteins involved in their activity. So, defects may regard the respiratory chain, with low production of ATP and loss of electrons with a corresponding increase of oxidative stress [1]. However, several studies have provided evidence that increased ROS production, particularly in the context of mitochondrial dysfunction, is not necessarily linked to ageing and enhancing antioxidant activity does not always lead to an increase in average lifespan [1]. In addition, low levels of ROS in mitochondria appear to have a beneficial hormetic effect, activating proliferative and survival pathways that support mitochondrial and cellular function. Consequently, the negative effects may depend on the sum of the already present damages induced by ageing [1].

The alteration of mitochondrial activity reflects on apoptotic processes, with increased membrane permeabilisation in response to stress and changes in lipidic composition, altered activation of inflammasome, and intracellular signalling, involving endoplasmic reticulum (ER) and, therefore, the protein post-translational and autophagy regulation [1].

Several studies regard the possibility to act on molecular pathways involved in mitochondrial dysfunction, including the modulation of proteins such as sirtuins, involved in the regulation of the mitochondrial activities. SIRT1 and 3 are the main ones involved in mitochondrial activity; the first acts on mitochondrial biosynthesis through a process involving the transcriptional peroxisome proliferator-activated receptor-γ coactivator (PGC-1α). The second one functions on the regulation of enzymes participating in metabolism and ROS production [1].

The role of OO and its compounds in supporting mitochondrial activity has been demonstrated in studies in which rats were fed OO throughout their lives [92–94]. The results were an enhanced expression of respiratory chain complexes, particularly coenzyme Q and cytochrome b, changes in the lipidic composition of the membrane associated with a decrease of hydroperoxide activity and an increase of catalase activity [92–94].

Direct evidence of the role of OLE and HT activity in response to ageing-induced alterations of mitochondria has also been evaluated on 3 T3-L1 adipocytes [25]. In this model, the addiction of HT at low concentrations (0.1–10 µmol/L) to the cultures promoted mitobiogenesis and enhanced mitochondrial functions. This effect was mediated by acting on PGC-1α and its downstream targets, including Nrf-1 and −2 and mitochondrial transcription factor A, which are all key regulators of mtDNA replication and biogenesis of organelles [25]. Furthermore, HT has been demonstrated to influence the expression of respiratory chain complexes. HT activates 5’-AMP-activate protein kinase, inducing increased expression of peroxisome proliferator–activated receptor -α and -γ and carnitine palmitoyltransferase 1. So, 48 h of HT stimulation took to the regulation of mitogenesis, β-oxidation factors, and fatty acids transport [25]. A 24-h pre-treatment with low doses of OLE led to the regulation of mitochondrial activity and biosynthesis, induction of apoptosis, regulation of membrane potential, and intracellular communication. This includes effects on ER post-translational activity and the elimination of unfolded proteins [26].

Conversely, some studies suggest that OLE and HT may exert detrimental effects on mitochondrial function. In particular, studies about the use of OO extracts on triple-negative breast and ovarian cancer cells have shown increased mitochondrial dysfunction, marked by reduced functionality, decreased membrane potential and antioxidant enzyme activity, along with elevated ROS levels. This oxidative effect could contribute to the anti-tumoral action of OLE and HT by highlighting the hormetic function of these compounds, with potential benefits or damage depending on the context and administered dose [24]. Higher doses of OLE or olive leaf extracts with elevated OLE concentration may also have toxic effects on normal cells, inhibiting cell growth, as described in the section above. On one hand, OLE and HT may stimulate mitochondrial biogenesis, with OLE specifically promoting mitochondrial fission by upregulating the expression of dynamin-related protein [69]. On the other hand, HT treatment in chondrocyte cultures appears to induce autophagy and mitophagy, likely mediated by the activation of SIRT1 [95].

The knowledge about OLE and HT in the treatment of mitochondrial dysfunction suggested a positive in the restoration of mitochondrial activity and functioning, paying attention to the hormetic properties of these compounds and the possibility of having opposite effects based on the concentration used.

## Effects of OLE and HT on loss of proteostasis

The concept of proteostasis regards the maintenance of protein homeostasis thanks to the action of chaperons, the ubiquitin–proteasome system and the autophagy processes. With ageing, there is an increase of chronicle pathologies associated with the accumulation of misfolded or aberrant proteins, which are accumulated inside cells instead of being removed, disrupting cellular homeostasis and thus impairing organ functions. Several studies pointed out the role of heat shock proteins (Hsp) in ageing advancement because of their involvement in the prevention of the accumulation of damaged proteins originating from stressor agents, like ROS. Indeed, it has been shown that the activation of Hs transcription factor (HSF)1, a transcriptional factor involved in the regulation of Hsp expression, determined the increased longevity in mice through the action of SIRT1. SIRT1 deacetylates HSF-1, and this determines the hyperactivation of Hsp70, a central chaperonin in the proteostasis process [96].

The altered clearance of proteins with ageing also involves the lysosomes, whose activity is impaired with ageing, and this has effects on the process of elimination of damaged cells, such as senescent ones [97]. Indeed, with ageing, an increase of lysosomal alkalinisation occurs [98]. This is associated with the acidification of the cytoplasm due to the accumulation of damaged proteins and to the alteration of lysosomal enzymes, which function optimally at acidic pH. This process accelerates cellular senescence and is linked to impaired mitochondrial function, increased inflammation, and ultimately, cell cycle arrest. An example of the dysfunction of lysosomes is given by the accumulation of toxic products like lipofuscin, which increases ROS production, caspase 3 and 1 activation, membrane disruption and inflammasome activation. This leads to impaired elimination of mitochondria, increased SASP, reduced apoptosis and deeper damage to cell functions [97]. The possibility of restoring the homeostatic conditions with direct intervention on the proteostatic levels may be a solution. EVOO secoiridoid-rich phenolic extract seems to act on the expression of several Hsp involved in the modulation of protein transport and regulation, like Hsp70 and Hsp72 [98]. Furthermore, OLE could increase proteasome activity, inhibiting the accumulation of aberrant proteins involved in the manifestation of diseases like Alzheimer. OLE seemed to determine the inhibition of toxic complexes formation and amyloid-β deposition reducing amyloid plaque formation in IMR90 and WI-38 embryonic fibroblasts and animal models of human muscles and Alzheimer-like pathology [44, 99]. Indeed, mice treated with 695 μg/kg/day of olive leaf extract, enriched with OLE, for 3 months showed a reduction of amyloid-β deposition and an inhibition of inflammasome [44]. Furthermore, OLE may act as a TFEB activator, a critical regulator of lysosome biogenesis. By promoting the fusion of autophagosomes with lysosomes, OLE facilitates the initiation of autophagic degradation processes [99]. Regarding autophagocytosis, OLE increases LC3II, p62, and Beclin (key proteins involved in autophagy) in in vitro experiments, through the inhibition of the mechanistic target of the rapamycin (mTOR)/protein kinase B (PKB or AKT) pathway, promoting the activation of AMP-activated protein kinase (AMPK) [27]. LC3 and Beclin are markers of autophagy activation, while p62 is involved in the recognition of the lysosome content [27]. AMPK represents the autophagosome inducer, and mTOR and mTORC1 are, instead, the inhibitor factors. A study on H_2_O_2_-mediated autophagic cell death in mesenchymal stem cells equally showed that the treatment with OLE decreased LC3 levels and the autophagy and apoptotic process induced by oxidative stimulus, inducing mTOR, Beclin, and the phosphorylation of AMPK [28]. The autophagy induction was also seen for a mice model of liver steatosis, demonstrating the positive effects of OLE regulating the mTOR pathway [45]. HT, then, also promoted autophagy markers by inducing SIRT1 expression [100].

## Effects of OLE and HT on deregulated nutrient-sensing pathways

The ageing process is often accompanied by metabolic changes in glucose and lipid metabolism, contributing to the onset of conditions such as obesity, increased insulin resistance and diabetes [101–103]. The molecular pathways involved in these processes have been identified as key markers of longevity, and their modulation in ageing models, often through dietary interventions, has emerged as a promising approach to extend both lifespan and health span. Central to the regulation of these metabolic pathways are the somatotropic hormone and its secondary effector, the insulin-like growth factor (IGF) [104, 105]. IGF responds to glucose levels, activating downstream signals similar to those triggered by insulin. For this reason, both IGF and insulin are integral components of the “insulin and IGF-1 signalling” (IIS) pathway [104].

Other key pathways comprising the nutrient sensing pathways (NSP) are mTOR, AKT, FOXO, AMPK, and sirtuins. FOXO overexpression in mice has been associated with enhanced lifespan, as well as the downregulation of mTOR/ribosomal protein S6 kinase beta-1 signalling and the upregulation of AMPK, consistent with its role in regulating the autophagy process. Conversely, during ageing, impaired cell function leads to low energy status and reduced levels of ATP. This energy deficit induces the activation of AMPK, which in turn inhibits mTOR and regulates every downstream signal related to mTOR inhibition, including the autophagy process [106]. In this contest, sirtuins, involved in several ageing hallmarks, are also a major factor in nutrient-sensing pathways. SIRT1, particularly, is involved in many mechanisms, stimulating mitobiogenesis and antioxidant activity [107].

The effects of OLE and HT on the dysregulation of NSP appear to be linked to the modulation of both the mTOR and AMPK pathways. In this regard, they may be considered as xenohormesis, functioning as slowing down ageing factors, activating the AMPK pathway. In this contest, they are considered as geroprotectors molecular pathways [98]. Furthermore, several animal studies have demonstrated that treatment with 20 mg/kg of OLE for 16 weeks, intraperitoneal injection of 20 mg/kg olive mill waste polyphenols for 3 months, and administration of 8–16 mg/kg OLE and HT-enriched extracts for 4 weeks resulted in reduced glucose levels, decreased oxidative stress, and improved insulin sensitivity, thus modulating the IIS [41, 42]. The same results were obtained with a human group of patients susceptible to insulin resistance, in which the treatment with olive leaf extract determined a greater insulin sensitivity and increased β-pancreatic isles functioning than the placebo group. The treatment with a capsule of olive extracts containing 51.1 mg OLE and 9.7 mg HT for 12 weeks, followed by a washout period, showed greater levels of IL-6 and IGF-binding protein-1 and −2 [50].

OLE and HT have effects on molecular pathways involved in the regulation of nutrient sensing and proteostasis, suggesting that the alteration found during the ageing process could be ameliorated through the administration of the correct concentration of these compounds. Indeed, nutrient-sensing pathways are integral to achieving longevity because they help maintain the organism’s metabolic balance, optimise energy usage, promote cellular repair, and activate stress-resistance mechanisms. By regulating processes such as autophagy, mitochondrial function, and DNA repair, these pathways enhance the ability to cope with ageing-related damage and reduce the risk of ARDs. Consequently, a well-regulated nutrient-sensing system contributes to a healthier, longer life [108].

## Effects of OLE and HT on cellular senescence

Cellular senescence is a physiological process triggered by several mechanisms, including telomere attrition, irreversible DNA damage and/or induced stressors [1, 2]. Cellular senescence induces a permanent cessation of cell division, leading to a stable, non-proliferative state marked by resistance to apoptosis [108]. This resistant pathway is defined by senescent cell anti-apoptotic pathways (SCAPs), defined by BCL-2/BCL-XL family members, P13 K/AKT, HIF-1α, and others [109–112]. This state is distinct from quiescence, a reversible form of cell cycle arrest, and terminal differentiation, in which cells cease division but acquire specialised functions [111]. Although SCs are in a non-replicative state, they remain metabolically active and secrete a complex mixture of factors known SASP [110–112]. SASP influences the tissue microenvironment by modulating inflammation, promoting the propagation of senescence, and exhibiting both protective and detrimental effects depending on the context [111]. Additionally, SASP demonstrate significant heterogeneity depending on the type of senescence inducer and the specific cell type affected, determining a strong variability also in the effects induced by SCs, such as the determination of the immune escape and the sprouting of senescence [113].

In this regard, OLE and HT appear to act as senomorphics, agents that suppress SASP activity, through the inhibition of NF-κB, without killing SCs (e.g., metformin, rapamycin) but reducing the mediators involved in their damage-induction. By modulating SCs and their secretory profile, senotherapeutic strategy holds promise for mitigating age-related pathologies and improving health span. OLE and HT, indeed, regulate the release of cytokines such as IL-6, TNF-α, and IL-10, and modulate chemokine expression, like RANTES and MCP-1, which are involved in the recruitment of immune cells, in this case macrophages [22]. Other stress-induced pathways involved in the rising of cellular senescence could be regulated by OLE and HT. As mentioned above, OLE regulates ROS production by acting on Nrf-2 [23], as well as on heme oxygenase (HO)—1, Hsp70, SIRT1, cyclooxygenase (COX)—2, inducible nitric oxide synthase (iNOS) and metalloproteinases (MMPs) involved in the previous features and contributing to the increase of senescence [22]. OLE and HT action in the inhibition of the senescence program could be evaluated by measuring the reduced the number of SA-β-Gal-positive cells. SA-β-Gal acts as a marker of senescence and permits the analysis of the levels of senescence [111]. In particular, a study conducted treating senescence-induced neonatal human dermal fibroblasts, with OLE aglycone (OLEa) 5 µM and HT 1 µM, showed a reduction due to the decreased number of cells with increased SA-β-Gal activity [22]. Furthermore, OLE and HT treatment had effects on senescence-induced DNA damage, preserving the integrity of the nuclear envelope, evaluated through lamin B1 expression and preventing the shedding of damaged chromatin from the nucleus to the cytoplasm. This last phenomenon is a trigger factor of pro-inflammatory cytokine production because of the activation of the cGAS-STING pathway, which drives interferon and other cytokine production [22].

Furthermore, it is plausible to suggest that phenolic compounds in OO may play a role in reversing not only the senescence program itself but also the immunosenescence process. This mechanism could be particularly important as immunosenescence is associated with difficulties in the elimination of SCs, contributing to the accumulation of SASP and subsequent tissue damage [114].

The application of senomorphic agents is a promising tool to counteract ageing and particularly senescence, but the use of natural compounds or other molecules must be considered carefully for time of administration and doses. Eliminating SCs despite their predominant phenotypes could provoke the elimination of favourable factors, which act in the repair of tissues or the resolution of increased cellular senescence or could impair other functions depending on the pathway considered.

## Effects of OLE and HT on stem cell exhaustion

The maintenance of stem cells’ proliferative capacity is essential for preserving tissue integrity and function. However, this regenerative potential declines with age due to a gradual exhaustion of stem cells, leading them to adopt a senescent phenotype, characterised by the changes outlined in the preceding paragraphs [1].

Ageing also induces significant alterations in the extracellular microenvironment (ECM), including the niches where stem cells reside [115]. These environmental changes contribute to the varied responses of stem cells to ageing. In some cases, this leads to a “detrimental” adaptation, in which stem cells exhibit changes similar to those seen in somatic cells, including hallmarks of ageing such as increased expression of cell cycle arrest genes (e.g., p16^INK4^ and p21), telomere attrition, and defects in nuclear lamina structure [115]. These alterations promote stem cell senescence, undermining their regenerative ability and accelerating tissue ageing. SCs create a pro-inflammatory milieu, which not only intensifies stem cell depletion through the secretion of SASP but also increases the risk of cancer with age [1]. Each molecular alteration contributes to a feedback loop that amplifies the senescent phenotype, further driving stem cell exhaustion. Importantly, both insufficient and excessive stem cell proliferation can be detrimental. While inadequate proliferation compromises tissue maintenance over time, unchecked proliferation can lead to the premature depletion of stem cell niches. This delicate balance is evident in studies of intestinal stem cells, where loss of quiescence leads to niche exhaustion and early ageing [1].

In mammalian systems, nutrient-sensing pathways, particularly mTOR and IGF-1, play pivotal roles in regulating stem cell activity [1]. Modulating these pathways can enhance proliferative capacity. However, their dysregulation may serve as an adaptive response to protect stem cells from overuse. Such adaptations can impair proliferative potential, disrupt proteostasis, and disturb energy metabolism. Additionally, other regulators like AMPK and SIRT1 are crucial in preserving stem cell function and ensuring long-term cellular homeostasis and longevity [116].

Ultimately, stem cell exhaustion reflects a loss of cellular plasticity that is essential for effective tissue repair. Successful regeneration requires a supportive microenvironment shaped by cytokines, growth factors, and extracellular matrix remodelling. These cues foster cellular de-differentiation and plasticity across various tissue compartments. Upon injury, some plastic cells can revert to a multipotent progenitor state. Experimental evidence shows that the transient expression of the OCT4, SOX2, KLF4, and MYC (OSKM) transcription factors can induce global de-differentiation by suppressing cell identity programs and enhancing cellular plasticity. For therapeutic regeneration, this process must be precisely controlled; turning “OSKM off” at the right stage allows re-differentiation and restoration of original cell identities [2, 117]. In this sense, OLE appears to directly influence the proliferative and differentiation potential of stem cells while indirectly contributing to the modulation of stem cell exhaustion by acting on ageing-related hallmarks involved in this phenomenon. A study clarified that the treatment with OLE 10^−6^ to 10^−4^ M induced osteoblastogenesis in human multipotential mesenchymal cells from bone marrow and had protective effects on bone loss during ageing, including the inhibition of osteoporosis [29]. The treatment of endothelial progenitor cells with OLE (obtained from leaves of *Ligustrum vulgare* L.) at the concentrations from 1.0 to 10.0 μM restored the capacity of migration and adhesion of these cells in a concentration-dependent manner before the treatment with angiotensin II (AngII). AngII, indeed, induces senescence and impairs the function of endothelial progenitor cells, which are directly involved in restoring ischaemic and injured vessels [23]. The OLE effect was related to the increase of telomerase activity, paired to Nrf-2 transcription factor activation and the increase of HO-1 expression, which led to the reduction of SCs and intracellular ROS [86]. In another study, the use of OLE showed an anti-angiogenic effect on endothelial progenitor cells and resident mature microvascular endothelial cells [77]. This demonstrates the possible effects of OLE in the modulation of stem cell capacity to proliferate and, thus, to regulate the related alteration linked to the ageing process.

Studies investigating the role of HT in modulating stem cell proliferation and differentiation are currently limited, with most focusing on cancer stem cells or the induction of specific differentiation programs. Notably, HT has been shown to stimulate the proliferation of stem and progenitor cells in the dentate gyrus of aged mice, promoting the generation and survival of new neurons [49]. In contrast, high concentrations of HT appear to exert inhibitory effects on osteogenic differentiation in human mesenchymal stem cells, suppressing the expression of osteoblastic markers. At the same time, these concentrations enhance adipogenic differentiation, as evidenced by increased expression of adipogenic genes and the formation of lipid vesicles [65].

In conclusion, OLE and HT demonstrate the capacity to modulate stem cell function and counteract features of stem cell exhaustion, albeit through distinct mechanisms. The claim that these compounds definitively inhibit stem cell exhaustion remains only partially substantiated and needs more studies. Indeed, OLE shows broader potential in restoring stem cell activity, reducing senescence, and supporting tissue-specific differentiation, particularly in bone and vascular systems [23, 77, 86]. HT, while less explored, contributes to neural regeneration and exhibits dose-sensitive effects on mesenchymal stem cell fate [30, 49]. Then, considering that stem cell exhaustion is affected by all the same changes observed in somatic cells during the ageing process, it is important to highlight that the mechanism of action of these compounds could also be interpreted in light of the involvement of several pathways associated with each of the previously described hallmarks of ageing. In this context, it was mention that OLE has a role in the inhibition of H_2_O_2_-induced autophagy and apoptosis in mesenchymal stem cells [28]. mTOR, which is involved in the activation of stem cell proliferation, prompting the exit from the quiescent status, is a possible target of OLE, as seen previously [45]. OLE exerts an inhibitory effect on mTOR, downregulating its activity and thereby facilitating the recovery of the autophagy process [45]. This highlights the complexity of targeting molecular pathways as interventions can yield both beneficial and adverse effects depending on the context. Therefore, it is crucial to precisely target the recipient of the intervention to ensure that the therapeutic benefits are maximised while minimising potential drawbacks. This precision approach underscores the importance of tailoring treatments to individual cellular and molecular dynamics [106].

## Effects of OLE and HT on altered intercellular communication

Communication between cells happens thanks to the release of soluble factors, like inflammatory mediators, or through direct contact thanks to gap junctions, which permit the exchange of cellular information [1]. As described by Lopez-Otin et al. [1], the main event in the alteration of intercellular communication is caused by inflammageing and the continuous release of cytokines and other SASP in the ECM. SCs practise a paracrine or endocrine-like effect on the nearby cells, determining the exacerbation of chronic conditions [111]. At the basis of the latent chronic inflammatory status, there is a broken homeostasis and balance between pro-inflammatory and anti-inflammatory pathways and between SCs and their clearance [111]. This status is strongly associated with the elevated activation of the NF-κB pathway and the impaired redox signalling pathways. Together with inflammation and oxidative stress, the altered autophagy process is linked to inflammasome activation and the expression of pro-inflammatory cytokines [118]. SIRT1, SIRT2, and SIRT6 involved in the dysregulation of nutrient sensing pathways and epigenetic modification, have a role in the regulation of NF-κB expression and inflammatory process [76]. Consequently, the inhibition of NF-κB has been demonstrated as a promising tool to restore the altered intracellular communication which occurs with ageing. Among the markers associated with inflammation, there are also hormones, like the gonadotropin-releasing hormone [119] and some miRNAs (i.e., miR-155, miR-21, and miR-146a) defined as inflamma-miRs [120, 121]. The inflamma-miRs increase in aged people, in association with pro-inflammatory markers, like C-reactive protein and fibrinogen and regulate the inflammatory pathways [122].

The alteration of intracellular communication, particularly the decrease in anti-inflammatory processes and the reduced release of pro-inflammatory mediators, is also driven by the impaired functionality of immune system cells [114]. These cells, affected by the process of immunosenescence, lose their ability to effectively eliminate SCs, either directly or indirectly. As a result, the body’s ability to counteract inflammation and maintain tissue homeostasis is compromised, contributing to the ageing-related decline in immune function. In this contest, the addiction of EVOO extracts, rich in OLEa and HT, showed action on bone marrow dendritic cells of mice, in terms of production of IL-6, TNF, IL-1β, iNOS, after lipopolysaccharide (LPS) stimulation, reducing the percentage of activated dendritic cells and the activation of inflammatory response by T cells [31].

Another effect associated with altered intercellular communication is the paracrine influence exerted by SCs through the exchange of damaging molecules via gap junctions. In co-culture with young fibroblasts, senescent fibroblasts induced senescence in the latter, as measured by double-strand breaks, likely caused by the passage of ROS through the gap junctions [123]. The bystander effect caused by DNA damage spreading from SCs has also been demonstrated in mouse models. In these models, senescent hepatic cells formed clusters of DNA-damaged cells [124]. Similarly, in a model of idiopathic pulmonary fibrosis, the transmission of the senescent phenotype was observed both through the conditioned medium of SCs and co-culture of senescent and non-SCs [125]. This resulted in increased mRNA levels of p21, IL-6, and IL-8, as well as the presence of γH2 AX, a marker of senescence-related and repair-resistant DNA double-strand breaks [123, 126–129]. The contribution of OLE and HT in these fields is still lacking in the literature, but presumably, it is possible to suppose a direct role also in intercellular communication.

## Effects of OLE and HT on the new hallmark of ageing

In recent years, additional hallmarks of ageing have been proposed López-Otín et al. [2]. These are not entirely new but are rather encompassed within the existing nine hallmarks defined by López-Otín et al. in 2013, as they function as part of broader ageing mechanisms [1]. These include inflammation, altered cell motility, dysregulation of DNA processing, microbiome disturbance and compromised autophagy [2]. They become predominant factors when viewed in the light of their pleiotropic contribution to the manifestation, progression and resolution of many age-related phenomena, starting with ARDs and cellular senescence. Inflammation and autophagy are already part of altered intercellular communication and loss of proteostasis hallmarks, respectively [1]. The altered mechanical properties of cellular components are related to cellular senescence and the incapacity to model the cytoskeleton components or to favour cell motility. For example, in fibroblasts, with ageing, the actin fibres become stable and anchored to the membrane, not permitting rapid polymerisation and depolymerisation; in immune cells, the altered expression of chemokines and their receptors induced wrong recruitment in the site of phlogosis, such as in the case of neutrophils in aged people [130]. OLE and HT influence the expression of chemokines in monocytes and macrophages, impacting the capacity of these cells to activate the T cell’s response and to respond to the stimuli. In an in vitro model of breast carcinoma, 0.01% OLE treatment for 2 h to 10 days induced disruption of the actin filament, suggesting a role of OLE in the modification of the cytoskeleton and the cellular motility capacity, particularly in the cancer cell lines [32].

The dysregulation of RNA processing during ageing involves alterations in epigenetic modifications, splicing processes, alternative polyadenylation, and the expression or activity of translational/transcription factors and miRNAs. Splicing, a post-transcriptional process that enhances proteome diversity by generating different proteins from the same genes, is particularly susceptible to ageing-related genomic instability and epigenetic changes [2, 131]. Ageing affects splicing sites and regulatory factors, such as enhancer or silencer proteins, and is linked to the misregulation of splicing regulators like the ATM gene in leukocytes, which encodes a key protein for DNA damage repair pathways (e.g., MYC, ARF, MDM2, p53). In vitro studies on fibroblasts, endothelial cells, and lymphocytes have shown that splicing alterations lead to tissue-specific heterogeneity in protein expression and impact the regulation of splicing factors, rendering transcripts susceptible to nonsense-mediated decay [132]. While OLE and HT modulate miRNAs involved in apoptosis, inflammation, and gene methylation, their role in splicing regulation remains unclear [78, 133].

Dysbiosis is a common characteristic of ageing. The modification of the microbiome is linked not only to ARDs but also to the onset of inflammageing [118]. The composition of the microbiome changes at the different stages of life, from infancy to old age, exhibiting increased diversity but decreased stability in its components [118]. An important extrinsic factor contributing to these changes is diet, as the feeding habits of the older ones differ from those of the young. Additionally, dysbiosis, which can be caused by medication use, further impacts the balance of the microbiome in ageing individuals [134]. The consumption of EVOO has been demonstrated as a protective factor for the maintenance of gut microbiota diversity and function, favouring the increment of *Clostridium* species and provoking the formation of butyrate, which has a role in the production of cholesterol and as an anti-inflammatory agent [135]. Furthermore, these bacteria are involved in the degradation of OLE, transforming it into a probiotic agent that performs its anti-inflammatory and antioxidant activity [135]. In a mouse model of diabetes, the administration of 200 mg/kg of OLE for 15 weeks has effects on the levels of fast glucose and glucose tolerance, impacting the phylum composition of the gut microbiota [48]. Once absorbed, lactic acid bacteria degrade OLE, and HT is the main product of its metabolisation by gut microbiota. In addition, several studies pointed out the potential antimicrobial effects of OLE and HT, which could modify the microbiota composition [136]. Indeed, OLE and HT are effective in the inhibition of *Enterobacteriaceae* growth, while HT can favour the growth of *Bifidobacteria* [137]. The activity of OLE appears to be concentration-dependent against some species, like *Staphylococcus aureus* and *Escherichia coli*, while lactic acid bacteria could be more resistant to OLE but not to HT [138].

Finally, the anti-inflammatory properties of HT and OLE are extensively studied and used in experimental models [9]. The capacity of HT-enriched OO to reduce inflammation, oedema, and bone dysfunction in a mouse model of rheumatoid arthritis has been shown [47]. The administration of OO contained 5 mg/kg of HT impacted on TNF-α serum levels, on iNOS and COX-2 expression [46]. In the same way, the treatment with HT-20, a preparation with 20% of HT, determined the reduction of TNF-α and IL-1β mRNA in a mouse model of acute inflammation and hyperalgesia, not influencing IL-10 levels [46]. Furthermore, THP-1 macrophages treated with increasing doses of HT (i.e., 25, 50, and 100 μM), before stimulation with LPS, showed a dose-dependent reduction of TNF-α, iNOS, and COX-2 at both protein and gene levels, highlighting the strong connection between the modulation of the inflammatory and oxidative processes [33]. Similar results were observed in inflammation models involving LPS-stimulated J774 murine macrophages and monocytes. In these models, the downregulation of iNOS and COX-2 gene expression was achieved by inhibiting the activation of NF-κB, STAT-1α, and IRF-1 [34]. Additionally, a reduction in mRNA levels of COX-2 and prostaglandin E2 was noted, along with an increase in TNF-α production [35]. These findings further highlight the potential of targeting inflammatory pathways to modulate immune responses. Furthermore, HT has been demonstrated to act in the modulation of other molecular pathways besides NF-κB, like JNK and p38 [36], inducing the phosphorylation and so the reduction of upstream signals in PBMCs treated with sterols compounds to simulate the pro-inflammatory and pro-oxidative process.

The treatment of THP-1 cell cultures with OO extracts, enriched in OLEa, modulated the levels of matrix MMP-9, TNF-α expression and also adhesion molecules like E-selectin, ICAM-1, and VCAM-1, through the modulation of NF-κB, in a model of vascular diseases like atherosclerosis, in which the inflammatory component is prevalent [37]. An effort to this study has been made by the administration of OLE to a myocardial infarction group of people, which has taken to the reduction of TNF-α and IL-1β levels [139].

Another study on the capacity of OO extracts to reduce the inflammation related to obesity demonstrated that OLEa acts on the monocyte differentiation, decreasing the levels of expression of CD16, on the capacity to recruit immune cells in the site of phlogosis, modulating chemokine expression, and on the expression of pro-inflammatory genes [38]. It is, therefore, possible to consider the use of OLE and HT as immunomodulators, capable of acting on molecular pathways involved in inflammation and oxidative stress, as well as reversing the cellular dysfunctions that affect immune cells during ageing [140].

## Geroprotective applications of OLE and HT

Acting at both molecular and metabolic levels, OLE and HT support the restoration of cellular homeostasis, potentially enhancing quality of life and reducing the incidence of chronic conditions, particularly those related to the ageing process [86]. Notably, their ability to modulate chronic inflammation and oxidative stress stands out [8, 9]. Their capacity to reduce pro-inflammatory cytokines and boost antioxidant defences suggests they could alleviate inflammageing and promote cellular homeostasis [9]. The findings underscore the multifaceted role of these compounds in targeting pathways involved in cellular senescence and metabolic regulation, two processes that play a critical role in the ageing process and the onset of ARDs [6, 9].

Research highlights the potential of OLE and HT to act not only as anti-inflammatory and antioxidant agents but also to influence the epigenome, autophagy, and mitochondrial function, all of which are central to the development of ageing [78]. The anti-senescence properties of OLE and HT, for example, may help delay or prevent the accumulation of dysfunctional cells, which are often involved in the onset of age-related conditions. Furthermore, their role in modulating metabolic pathways could offer protection against metabolic diseases such as diabetes, obesity, and insulin resistance, which often exacerbate the ageing process [50].

However, despite promising results from in vitro and in vivo studies, further robust clinical trials are needed to assess their efficacy in humans. While preliminary studies show beneficial effects, the complex nature of ageing requires well-controlled, long-term trials to fully understand the mechanisms and outcomes of these interventions in ageing populations. Moving forward, research should focus not only on evaluating the geroprotective properties of these compounds in clinical settings but also on understanding how they work in vivo. Investigating their effects in human trials will provide crucial insights into dosage, timing, and potential synergistic effects when combined with other therapeutic agents.

An exciting prospect for OLE and HT lies in their potential integration as adjuvants or additives in vaccines and drug formulations. By enhancing immune responses and reducing the inflammatory burden, they could reduce the onset of ARDs and mitigate therapy-related side effects derived from inflammageing and immunosenescence rising with ageing. Ongoing clinical trials are investigating the use of OLE and other phenolic compounds as vaccine adjuvants, particularly for influenza and other diseases, aiming to develop more effective, sustainable, and eco-friendly pharmaceutical solutions. Whether used as dietary supplements or incorporated into pharmaceutical formulations, OLE and HT could significantly contribute to extending both lifespan and health span. Understanding the appropriate dosages and long-term effects of OLE and HT is essential for their successful application as geroprotective agents [98]. Additionally, the potential hormetic effects of OLE and HT warrant careful consideration, as their benefits may vary significantly depending on concentration, duration of exposure, and the specific biological context [98]. Suboptimal concentrations could stimulate cellular repair mechanisms, stress resistance pathways, and anti-inflammatory processes without causing harm, while higher concentrations may become cytotoxic or trigger undesirable effects, such as excessive oxidative stress or immune suppression. The precise mechanisms underlying the hormetic effects of OLE and HT need to be better understood, as they could offer a way to harness the protective power of these compounds without overloading the system. For instance, at low to moderate doses, HT and OLE may enhance mitochondrial function, autophagy, and antioxidant defences, providing protection against cellular damage and promoting healthy ageing. However, at higher concentrations, they could exacerbate inflammatory pathways or disrupt normal cellular signalling, potentially counteracting the very benefits they offer at lower doses [98].

Possible therapeutic applications involving OLE and HT could fear using these compounds to increase the production of exosomes by target cells. Exosomes are vesicles which transport molecules produced by the cells, enabling cellular communication, thus mediating, for example, paracrine or endocrine effects to nearby cells [141]. The study of exosomes is extensive in terms of diagnostics, but the idea of using these vesicles for therapeutic personalised and tailored therapy is appealing as well [142]. A recent study (Mantilla-Escalante et al., 2021) highlighted how 1-year administration of a MedDiet rich in EVOO or nuts, to men aged 55–80 years and women aged 60–80 years, without CVDs but with at least three cardiovascular risk factors and with diabetes, increases the modulation of miRNAs present at the level of exosomes [143]. The targets of miRNAs involved are those that determine various biological pathways, including those concerning hallmarks of ageing, such as NF-кB, AKT, p53, and others [143].

In addition, exosomes are recognised as products of SCs, capable of spreading the senescent phenotype to neighbouring cells, carrying factors that promote the onset of cellular senescence. Then, their production is found to be increased in different models of cellular senescence, underscoring their importance in this context [141]. The ability to target and even manipulate exosomes via EVOO compounds could fall into the class of geroprotective therapies and open to other suggestions about the use of these compounds indirectly on hallmarks of ageing. Additionally, evaluating how these interventions may interact with existing preventive strategies, particularly those aimed at protecting the oldest ones from infections, chronic diseases, and cognitive decline, is critical. It is important to explore how OLE and HT might enhance the efficacy of vaccines, antibiotic treatments, or immune boosters, making them valuable allies in geriatric medicine. The successful integration of OLE and HT into clinical practice could represent a paradigm shift in age-related healthcare, offering a natural, safe, and potent approach to improving both the quality of life and health outcomes for ageing populations worldwide.

## Conclusion

This review highlights the growing interest in natural compounds as promising strategies for ageing intervention, opening the door for further investigation into their mechanisms and therapeutic potential. The identification of natural compounds capable of slowing down or even reversing some of ageing changes offers a hopeful pathway for the development of novel therapies for the oldest individuals. These compounds have demonstrated a significant impact on critical ageing processes, including genomic instability, proteostasis disruption, cellular senescence, mitochondrial dysfunction, and chronic low-grade inflammation (inflammageing), all of which are essential for maintaining tissue integrity and delaying the onset of ARDs.

So, recognition and expansion of the research on OLE and HT as geroprotectors could help mitigate ageing-related alterations, as their effects on the hallmarks of ageing have been thoroughly evaluated.

## Data Availability

Not applicable.
